# Lopinavir plus nucleoside reverse-transcriptase inhibitors, lopinavir plus raltegravir, or lopinavir monotherapy for second-line treatment of HIV (EARNEST): 144-week follow-up results from a randomised controlled trial

**DOI:** 10.1016/S1473-3099(17)30630-8

**Published:** 2018-01

**Authors:** James G Hakim, Jennifer Thompson, Cissy Kityo, Anne Hoppe, Andrew Kambugu, Joep J van Oosterhout, Abbas Lugemwa, Abraham Siika, Raymond Mwebaze, Aggrey Mweemba, George Abongomera, Margaret J Thomason, Philippa Easterbrook, Peter Mugyenyi, A Sarah Walker, Nicholas I Paton, Europe Africa Research Network for Evaluation of Second-line Therapy (EARNEST) Trial Team, E Agweng, P Awio, G Bakeinyaga, C Isabirye, U Kabuga, S Kasuswa, M Katuramu, F Kiweewa, H Kyomugisha, E Lutalo, D Mulima, H Musana, G Musitwa, V Musiime, M Ndigendawan, H Namata, J Nkalubo, P Ocitti Labejja, P Okello, P Olal, G Pimundu, P Segonga, F Ssali, Z Tamale, D Tumukunde, W Namala, R Byaruhanga, J Kayiwa, J Tukamushaba, S Abunyang, D Eram, O Denis, R Lwalanda, L Mugarura, J Namusanje, I Nankya, E Ndashimye, E Nabulime, D Mulima, O Senfuma, G Bihabwa, E Buluma, A Elbireer, D Kamya, M Katwere, R Kiggundu, C Komujuni, E Laker, E Lubwama, I Mambule, J Matovu, A Nakajubi, J Nakku, R Nalumenya, L Namuyimbwa, F Semitala, B Wandera, J Wanyama, H Mugerwa, E Ninsiima, T Ssenkindu, S Mwebe, L Atwine, H William, C Katemba, S Abunyang, M Acaku, P Ssebutinde, H Kitizo, J Kukundakwe, M Naluguza, K Ssegawa, Namayanja, F Nsibuka, P Tuhirirwe, M Fortunate, J Acen, J Achidri, A Amone, M Chamai, J Ditai, M Kemigisa, M Kiconco, C Matama, D Mbanza, F Nambaziira, M Owor Odoi, A Rweyora, G Tumwebaze, H Kalanzi, J Katabaazi, A Kiyingi, M Mbidde, M Mugenyi, P Okong, I Senoga, M Abwola, D Baliruno, J Bwomezi, A Kasede, M Mudoola, R Namisi, F Ssennono, S Tuhirwe, G Amone, J Abach, I Aciro, B Arach, P Kidega, J Omongin, E Ocung, W Odong, A Philliam, H Alima, B Ahimbisibwe, E Atuhaire, F Atukunda, G Bekusike, A Bulegyeya, D Kahatano, S Kamukama, J Kyoshabire, A Nassali, A Mbonye, T M Naturinda, Ndukukire, A Nshabohurira, H Ntawiha, A Rogers, M Tibyasa, S Kiirya, D Atwongyeire, A Nankya, C Draleku, D Nakiboneka, D Odoch, L Lakidi, R Ruganda, R Abiriga, M Mulindwa, F Balmoi, S Kafuma, E Moriku, A Reid, E Chidziva, G Musoro, C Warambwa, G Tinago, S Mutsai, M Phiri, S Mudzingwa, T Bafana, V Masore, C Moyo, R Nhema, S Chitongo, Robert Heyderman, Lucky Kabanga, Symon Kaunda, Aubrey Kudzala, Linly Lifa, Jane Mallewa, Mike Moore, Chrissie Mtali, George Musowa, Grace Mwimaniwa, Rosemary Sikwese, Milton Ziwoya, H Chimbaka B Chitete, S Kamanga, T Kayinga E Makwakwa, R Mbiya, M Mlenga, T Mphande, C Mtika, G Mushani, O Ndhlovu, M Ngonga, I Nkhana, R Nyirenda, P Cheruiyot, C Kwobah, W Lokitala Ekiru, M Mokaya, A Mudogo, A Nzioka, M Tanui, S Wachira, K Wools-Kaloustian, P Alipalli, E Chikatula, J Kipaila, I Kunda, S Lakhi, J Malama, W Mufwambi, L Mulenga, P Mwaba, E Mwamba, M Namfukwe, E Kerukadho, B Ngwatu, J Birungi, J Boles, A Burke, L Castle, S Ghuman, L Kendall, S Tebbs, J Whittle, H Wilkes, N Young, M Spyer, C Kapuya, F Kyomuhendo, D Kyakundi, N Mkandawire, S Mulambo, S Senyonjo, B Angus, A Arenas-Pinto, A Palfreeman, F Post, D Ishola, J Arribas, R Colebunders, M Floridia, M Giuliano, P Mallon, P Walsh, M De Rosa, E Rinaldi, I Weller, C Gilks, A Kangewende, S Lakhi, E Luyirika, F Miiro, S Ojoo, S Phiri, A Wapakabulo, T Peto, J Matenga, G Cloherty, J van Wyk, M Norton, S Lehrman, P Lamba, K Malik, J Rooney, W Snowden, J Villacian

**Affiliations:** a University of Zimbabwe Clinical Research Centre, Harare, Zimbabwe; b MRC Clinical Trials Unit at University College London, London, UK; c Joint Clinical Research Centre (JCRC) Kampala, Kampala, Uganda; d Infectious Diseases Institute, Kampala, Uganda; e Department of Medicine, University of Malawi College of Medicine, Blantyre, Malawi; f Dignitas International, Zomba, Malawi; g JCRC Mbarara, Mbarara, Uganda; h Moi University School of Medicine, Eldoret, Kenya; i St Francis of Nsambya Hospital, Kampala, Uganda; j University Teaching Hospital, Lusaka, Zambia; k Yong Loo Lin School of Medicine, National University of Singapore, Singapore, Singapore

## Abstract

**Background:**

Millions of HIV-infected people worldwide receive antiretroviral therapy (ART) in programmes using WHO-recommended standardised regimens. Recent WHO guidelines recommend a boosted protease inhibitor plus raltegravir as an alternative second-line combination. We assessed whether this treatment option offers any advantage over the standard protease inhibitor plus two nucleoside reverse-transcriptase inhibitors (NRTIs) second-line combination after 144 weeks of follow-up in typical programme settings.

**Methods:**

We analysed the 144-week outcomes at the completion of the EARNEST trial, a randomised controlled trial done in HIV-infected adults or adolescents in 14 sites in five sub-Saharan African countries (Uganda, Zimbabwe, Malawi, Kenya, Zambia). Participants were those who were no longer responding to non-NRTI-based first-line ART, as assessed with WHO criteria, confirmed by viral-load testing. Participants were randomly assigned to receive a ritonavir-boosted protease inhibitor (lopinavir 400 mg with ritonavir 100 mg, twice per day) plus two or three clinician-selected NRTIs (protease inhibitor plus NRTI group), protease inhibitor plus raltegravir (400 mg twice per day; protease inhibitor plus raltegravir group), or protease inhibitor monotherapy (plus raltegravir induction for first 12 weeks, re-intensified to combination therapy after week 96; protease inhibitor monotherapy group). Randomisation was by computer-generated randomisation sequence, with variable block size. The primary outcome was viral load of less than 400 copies per mL at week 144, for which we assessed non-inferiority with a one-sided α of 0·025, and superiority with a two-sided α of 0·025. The EARNEST trial is registered with ISRCTN, number 37737787.

**Findings:**

Between April 12, 2010, and April 29, 2011, 1837 patients were screened for eligibility, of whom 1277 patients were randomly assigned to an intervention group. In the primary (complete-case) analysis at 144 weeks, 317 (86%) of 367 in the protease inhibitor plus NRTI group had viral loads of less than 400 copies per mL compared with 312 (81%) of 383 in the protease inhibitor plus raltegravir group (p=0·07; lower 95% confidence limit for difference 10·2% *vs* specified non-inferiority margin 10%). In the protease inhibitor monotherapy group, 292 (78%) of 375 had viral loads of less than 400 copies per mL; p=0·003 versus the protease inhibitor plus NRTI group at 144 weeks. There was no difference between groups in serious adverse events, grade 3 or 4 adverse events (total or ART-related), or events that resulted in treatment modification.

**Interpretation:**

Protease inhibitor plus raltegravir offered no advantage over protease inhibitor plus NRTI in virological efficacy or safety. In the primary analysis, protease inhibitor plus raltegravir did not meet non-inferiority criteria. A regimen of protease inhibitor with NRTIs remains the best standardised second-line regimen for use in programmes in resource-limited settings.

**Funding:**

European and Developing Countries Clinical Trials Partnership (EDCTP), UK Medical Research Council, Instituto de Salud Carlos III, Irish Aid, Swedish International Development Cooperation Agency, Instituto Superiore di Sanita, Merck, ViiV Healthcare, WHO.

## Introduction

Over 17 million people currently receive antiretroviral therapy (ART) for HIV infection worldwide, most of whom live in resource-limited settings. ART is usually delivered using the WHO-recommended public health approach, characterised by use of standardised sequential regimens and simplified monitoring and care.^1^ WHO-recommended standardised second-line therapy comprises two nucleoside reverse-transcriptase inhibitors (NRTIs) combined with a boosted protease inhibitor.^2^

There are theoretical reasons why replacing the NRTIs with raltegravir in second-line therapy might be advantageous, primarily the absence of cross-resistance to first-line therapy. Three randomised controlled trials^3^, ^4^, ^5^ have evaluated the combination of a protease inhibitor with raltegravir and did not show a benefit over standard protease inhibitor plus NRTI regimens after 48–96 weeks of follow-up, although all trials reported virological non-inferiority over this duration. Protease inhibitor plus raltegravir is included as an alternative regimen in the 2016 WHO treatment guidelines (with ritonavir-boosted lopinavir as the protease inhibitor).^2^ However, before changing the standardised WHO second-line regimens in large-scale treatment programmes, it is essential to evaluate the durability of this combination over a longer period than 48–96 weeks, and to investigate whether there are specific patient groups in which it has advantages or disadvantages.

**Research in context**

**Evidence before this study**

We searched PubMed using terms including “second-line therapy”, “protease inhibitors”, and the individual drug names, and reviewed relevant HIV conference abstracts to identify clinical trials done in patients who had failed on a first-line non-nucleoside reverse-transcriptase inhibitor (NNRTI)-based combination, which compared the standard-of-care protease inhibitor plus NRTI combination for second-line therapy with either a protease inhibitor plus raltegravir combination or with protease inhibitor monotherapy. No language or date restrictions were used. No relevant studies were identified in our initial search on March 1, 2009. An updated search on June 1, 2017, using the same terms, identified three published randomised controlled trials reporting outcomes after 48 or 96 weeks' treatment with the protease inhibitor plus raltegravir combination (including the earlier report from this trial) that concluded that this option was non-inferior, and two randomised controlled trials reporting outcomes after 48 or 96 weeks' treatment with protease inhibitor monotherapy (including the earlier report from this trial), which concluded that this option was inferior to standard of care.

**Added value of this study**

This trial provides the first comparative, randomised data on long-term (144 weeks) outcomes with the protease inhibitor plus raltegravir regimen in second-line therapy. With this longer duration of follow-up, we found that non-inferiority was not consistently demonstrated across all analyses, and there was no evidence of a safety benefit with the protease inhibitor plus raltegravir regimen compared with the standard-of-care protease inhibitor plus NRTI combination. The trial also confirms that the initial response of the protease inhibitor plus NRTI regimen is durable with longer-term follow-up and that reintroduction of combination therapy restores virological suppression after prolonged protease inhibitor monotherapy, emphasising the contribution of NRTIs to second-line regimen activity.

**Implications of all the available evidence**

Taking into account the higher cost of raltegravir, the absence of clear advantages of protease inhibitor plus raltegravir seen in any trial and the failure to show non-inferiority consistently across all analyses after 144 weeks of treatment in this trial suggest that there is no compelling reason for national programmes to adopt this combination as the standardised second-line therapy in the public health approach to antiretroviral therapy.

Here, we report outcomes after 144 weeks of follow-up from the EARNEST trial, the largest of the trials assessing protease inhibitor plus raltegravir and the only trial with follow-up beyond 96 weeks, and examine its performance in subgroups relevant for resource-limited settings. We also report outcomes after reintroduction of NRTIs after an initial period of protease inhibitor monotherapy as second-line treatment.

## Methods

### Study design and participants

The EARNEST trial was a randomised controlled trial done in 14 sites in five sub-Saharan African countries (Uganda, Zimbabwe, Malawi, Kenya, and Zambia). Eligible patients were HIV-infected adults or adolescents older than 12 years who were no longer responding to a first-line combination of NRTIs with a non-NRTI (based on WHO clinical, immunological, or virological criteria; all confirmed by viral load of >400 copies per mL).^3^ Exclusion criteria were pregnancy or breastfeeding, life expectancy of less than 1 month, contraindications to any of the study drugs, ongoing requirement for treatment with concomitant drugs with known interaction with any study drug, or known hepatitis B surface antigen positivity.

The protocol was approved by ethics committees in all participating countries and the UK. All participants (and caregivers of adolescents younger than 18 years) provided written informed consent.

### Randomisation and masking

Patients were randomly assigned (1:1:1) to one of three treatment groups. Randomisation was stratified by centre, and screening CD4 count (<200 cells per μL *vs* ≥200 cells per μL). The computer-generated, sequentially numbered randomisation list (variable block sizes) was preprepared by the trial statistician and incorporated in the online secure database. Randomisation was done by the trial manager at each centre, who could access the next number, but not the whole list. Treatment was given open-label (ie, patients and investigators were not blinded to treatment allocation).

### Procedures

Patients were randomly assigned a ritonavir-boosted protease inhibitor (standardised to lopinavir 400 mg and ritonavir 100 mg, both taken twice per day) plus two NRTIs (protease inhibitor plus NRTI group), plus raltegravir 400mg twice per day (protease inhibitor plus raltegravir group), or alone as monotherapy (after an initial 12-week raltegravir induction; protease inhibitor monotherapy group) and were followed up for 144 weeks. In the protease inhibitor plus NRTI group, NRTIs were selected by the physician without resistance testing, following WHO algorithms (switch to tenofovir if previously on stavudine or zidovudine, and vice versa) and taking into account side-effects on first-line, local standard-of-care, and local drug availability. The protocol initially allowed treatment with three NRTIs in Malawi (reduced to two NRTIs later when local guidelines changed). After a data monitoring committee (DMC) recommendation based on poorer viral-load suppression and more protease inhibitor resistance in the protease inhibitor monotherapy group than in the protease inhibitor plus NRTI group, patients in the protease inhibitor monotherapy group were switched to combination therapy (usually by reintroducing NRTIs) after May, 2013 (all patients had completed at least 96 weeks of follow-up after randomisation).

Patients were assessed every 4–8 weeks, with most visits done by nurses. Adherence was assessed at each visit through structured questions, with intensive adherence counselling when lapses were identified. Treatment was monitored clinically, with full blood count, alanine transaminase, serum creatinine, and urine dipstick tests for glucose, protein, and leucocytes done at weeks 12, 48, 96, and 144 after randomisation, and CD4 cell counts every 12–16 weeks in the local site's laboratory. Additional tests were permitted at the discretion of the treating clinician to evaluate and monitor incident adverse events. Within-class ART substitutions were allowed for toxicity or poor tolerability. There was no real-time viral-load monitoring, but if a patient developed clinical or immunological failure (definitions as for trial entry), and an alternative regimen was available locally, sites could do open local viral-load testing (and subsequent resistance testing, if it would affect drug selection) after approval from a clinical expert review committee (CERC) and change treatment if needed. In the protease inhibitor monotherapy group, after the recommendation by the DMC to reintroduce combination treatment, all trial viral-load and resistance testing results were provided to the treating clinicians who were permitted to additionally test for viral load and resistance in these patients at their discretion. Women assigned protease inhibitor monotherapy who became pregnant added NRTIs while pregnant or breastfeeding. Tuberculosis was treated using rifabutin, with ART unchanged.

During the trial, viral load was measured centrally (Joint Clinical Research Centre [JCRC], Kampala, Uganda) in batches of samples stored at weeks 48 and 96 using the Abbott RealTime HIV-1 assay (Abbott Laboratories, IL, USA); individuals who did the assays were blinded to randomised allocation. After trial closure, viral-load testing was done on samples stored at weeks 4, 12, 24, 36, 64, 80, 110, 126, and 144 (for protease inhibitor monotherapy, intermediate timepoints were tested systematically to week 48 only). Genotyping (reverse transcriptase, protease, and integrase, according to group) was done blinded on all post-randomisation samples with viral load of more than 1000 copies per mL at Janssen Diagnostics (Beerse, Belgium). Genotyping (reverse transcriptase) of baseline samples from patients in the protease inhibitor plus NRTI group and protease inhibitor plus raltegravir group was done at the JCRC (Kampala). Drug susceptibility prediction used the Stanford algorithm (version 7). Subtype was determined using REGA (version 3.0). Viral loads and genotypes were reviewed by the DMC, but not provided to treating clinicians during the trial (with the exception of relevant viral load and genotypes from protease inhibitor monotherapy group after the decision to reintensify therapy).

Neurocognitive function and peripheral neuropathy were assessed using standard methods.^6^, ^7^ The CERC, comprising four independent HIV physicians, adjudicated event reports against standard prespecified diagnostic criteria (stage 3 and 4 events, WHO criteria;^8^ serious non-AIDS events, INSIGHT criteria;^9^ adverse events, DAIDS criteria^10^) and assessed relationship to antiretroviral drugs.

### Outcomes

The primary endpoint for the 96-week analysis,^3^ reported elsewhere, was a composite endpoint (good disease control) based on clinical status through 96 weeks (no new WHO stage 4 events after randomisation other than oeosophageal candidiasis or mucosal herpes simplex virus infection), and CD4 count higher than 250 cells per μL, and viral load less than 10 000 copies per mL (or >10 000 copies per mL without major or minor protease inhibitor resistance mutations) at week 96. The primary outcome for the week-144 analysis was viral load of less than 400 copies at week 144. Secondary endpoints at week 144 were viral load of less than 50 copies per mL, viral load of less than 1000 copies per mL, protease inhibitor resistance mutations, intermediate or high level of lopinavir resistance, good disease control, survival, WHO stage 4 events, CD4 count greater than 250 cells per μL, CD4 count change from baseline, serious adverse events, grade 3 or 4 adverse events (all, or ART-related), and change in neurocognitive function from baseline.

### Statistical analysis

For the sample size calculation, we assumed that 75% of patients in the protease inhibitor plus NRTI group would have good disease control at week 96 and 10% would be lost to follow-up. With a one-sided α of 0·025 for the non-inferiority comparison and two-sided α of 0·025 for the superiority comparison, we estimated that 400 patients per group provided 80% power to show non-inferiority of protease inhibitor monotherapy using a 10% non-inferiority margin, and 87% power to demonstrate superiority of protease inhibitor plus raltegravir assuming 10% greater response rate.^3^

All comparisons were according to randomised arm (intention-to-treat) regardless of ART changes after randomisation. Statistical tests were two-sided and did not adjust for multiplicity. 95% CIs correspond to a two-sided test for superiority; for non-inferiority comparisons, the focus was on the lower confidence limit. The endpoint of good disease control used multiple imputations to account for missing CD4 cell count, viral loads, and genotypes (<5% of observations). Following the statistical analysis plan, the primary analysis of all other endpoints used complete-case analyses and excluded deaths, individuals lost to follow-up, and missed visits. Binary endpoints were compared using risk differences and χ^2^ tests, and continuous variables using mean change from baseline and *t* tests or ANOVA. Time-to-event endpoints were analysed using Cox proportional hazards regression and Kaplan-Meier. Generalised estimating equations (independent correlation structure, binomial for viral-load suppression and eGFR <60 mL/min per 1·73 m^2^, and normal distribution for CD4 and eGFR change) were used to test difference between groups across all visit weeks. Prevalence of intermediate-high level resistance at week 144 was adjusted for failed or missing genotypes using sampling weights.

Of three protocol-specified viral-load thresholds (50, 400, and 1000 copies per mL), we selected 400 copies per mL as the main outcome because it is less affected by transient, low-level viral-load blips, is closest to failure thresholds used in most contemporary treatment guidelines (although WHO uses a threshold of 1000 copies per mL),^2^, ^11^, ^12^, ^13^ and was the main outcome reported for viral-load suppression analyses at week 96.^3^ For the protease inhibitor plus NRTI versus protease inhibitor plus raltegravir comparison, in addition to the primary complete-case analysis, we also did several exploratory analyses of virological responses commonly used for industry trials, based on modified US Food and Drug Administration definitions. A per-protocol analysis excluded any patient who moved off their randomised ART strategy before week 144. A time to loss of virological response (TLOVR) analysis assigned an outcome of virological failure to those individuals who had no viral loads below the specified threshold up to and including week 24 (and had at least two viral loads in this period); and those who had confirmed viral load above the specified threshold (two consecutive viral loads, using the date of the first viral load as time of failure or the date of the first missing scheduled viral load after the preceding suppressed sample, if the confirmed viral load was preceded by missing viral loads), or switched treatment for failure (clinical or immunological, confirmed by real-time viral-load testing), or who had withdrawn or were lost to follow-up or died before week 144 or who had a missing viral-load test result at week 144. A snapshot analysis assigned an outcome of virological failure to patients who had a viral load above the specified threshold at week 144, who switched for failure (defined as above), who had withdrawn or were lost to follow-up or died before week 144, or who had a missing viral-load measurement at week 144.

Subgroup analyses comparing protease inhibitor plus NRTI versus protease inhibitor plus raltegravir groups at week 144 were done following the intention-to-treat principle, on the complete-case population using the same non-inferiority margin of 10% to compare the two treatment groups, although we recognise that the power to determine non-inferiority is lower within subgroups and the trial was not formally powered for this (additional details in the appendix p 3). All authors vouch for the completeness of the data and analyses presented and fidelity of this report to the protocol.

The EARNEST trial is registered with ISRCTN, number 37737787.

### Role of the funding source

The funder of the study had no role in study design, data collection, data analysis, data interpretation, or writing of the report. The corresponding author had full access to all the data in the study and had final responsibility for the decision to submit for publication.

## Results

**Figure 1:**
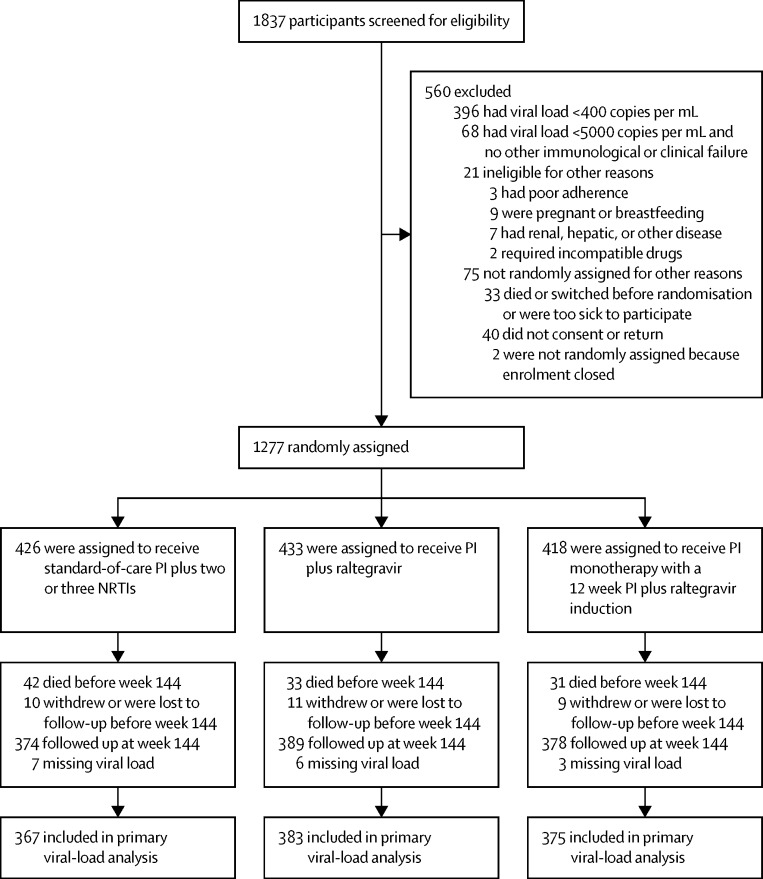
Trial profile NRTIs=nucleoside reverse-transcriptase inhibitors. PI=protease inhibitor.

Between April 12, 2010, and April 29, 2011, 1837 patients were screened for eligibility, of whom 1277 patients were randomly assigned to an intervention group. 426 patients were assigned to protease inhibitor plus NRTI, 433 patients were assigned to protease inhibitor plus raltegravir, and 418 patients were assigned to protease inhibitor monotherapy (figure 1); baseline characteristics were similar across treatment groups (table 1).^3^ Patients had advanced first-line treatment failure with high viral load (median 69 782 copies per mL; 530 [42%] of 1277 patients had viral loads of >100 000 copies per mL) and low CD4 count (median 71 cells per μL; 787 [62%] of 1277 patients had counts of <100 cells per μL), and extensive baseline resistance (769 [98%] of 787 patients had one or more major NRTI mutations).^14^ In the protease inhibitor plus NRTI group, 336 (79%) of 426 patients received tenofovir in their initial second-line regimen (all with lamivudine or emtricitabine; 37 [9%] patients had zidovudine as a third NRTI). By 144 weeks, 106 (8%) of 1277 patients had died, 30 (2%) had withdrawn or were lost to follow-up, and ten (1%, two patients in the protease inhibitor plus NRTI group, eight in the protease inhibitor monotherapy group) had switched ART due to treatment failure (figure 1).

**Table 1:** Baseline characteristics

		**PI plus NRTI (n=426)**	**PI plus raltegravir (n=433)**	**PI monotherapy (n=418)**
Sex				
	Male	162 (38%)	170 (39%)	203 (49%)
	Female	264 (62%)	263 (61%)	215 (51%)
Age (years)		37 (31–43)	37 (30–43)	38 (32–44)
	Range	12–73	12–75	12–71
BMI (kg/m^2^)		20 (18–23)	21 (18–23)	21 (18–23)
Known to be WHO stage 4		85 (20%)	98 (23%)	97 (23%)
CD4 count (cells per μL)		72 (29–143)	70 (27–142)	70 (33–149)
	<100	262 (62%)	267 (62%)	258 (62%)
Viral load (copies per mL)		67 515 (23 065–175 800)	74 500 (25 004–205 000)	70 874 (21 584–210 000)
	≥100 000	168 (39%)	181 (42%)	181 (43%)
ART history				
	Years on combination ART	4·0 (2·8–5·4)	4·0 (2·9–5·5)	3·9 (2·6–5·4)
Ever taken as first-line drug				
	Zidovudine	292 (69%)	283 (65%)	287 (69%)
	Stavudine	266 (62%)	266 (61%)	245 (59%)
	Tenofovir	52 (12%)	71 (16%)	60 (14%)
Laboratory parameters				
	Haemoglobin (g/dL)	11·9 (2·2)	11·9 (2·2)	12·0 (2·1)
	eGFR (mL/min per 1·73 m^2^)	114·7 (37·6)	114·8 (39·1)	112·5 (38·0)

Data are n (%), median (IQR), or mean (SD), unless otherwise specified. BMI calculated for 1243 (97%) participants with height available (one patient in the PI plus raltegravir group missing weight). WHO stage available for 766 (60%) participants: remainder came from other clinics without good previous medical history. Haemoglobin available for 1268 (99%) participants and eGFR available for 1238 (97%) participants. Additional baseline characteristics have been published previously.^3^ PI=protease inhibitor. NRTI=nucleoside reverse-transcriptase inhibitors. BMI=body-mass index. ART=antiretroviral therapy. eGFR=estimated glomerular filtration rate.

At week 144, 317 (86%) of 367 participants in the protease inhibitor plus NRTI group had viral loads of less than 400 copies per mL, 276 (75%) had viral loads of less than 50 copies per mL, and 321 (87%) had viral loads of less than 1000 copies per mL (table 2). At week 144, intermediate or high-level resistance to one or more NRTIs (excluding lamivudine and emtricitabine) taken during the trial was seen in ten participants (3% of protease inhibitor plus NRTI group overall, adjusting for failed genotypes) and to lopinavir in seven patients (2%, one with intermediate-level cross-resistance to darunavir; table 2).

**Table 2:** Viral load, resistance, and main efficacy outcomes at 144 weeks

		**PI plus NRTI (n=426)**	**PI plus raltegravir (n=433)**	**PI monotherapy (n=418)**	**Global p value**	**PI plus raltegravir *vs* PI plus NRTI**		**PI monotherapy *vs* PI plus NRTI**	
						Risk difference and HR (95% CI)*	p value	Risk difference and HR (95% CI)*	p value
Viral load (copies per mL)									
	Available	367	383	375	··	··	··	··	··
	<50	276 (75%)	275 (72%)	246 (66%)	0·01	−3·4% (−9·7 to 2·9)	0·29	−9·6% (−16·1 to −3·1)	0·004
	<400	317 (86%)	312 (81%)	292 (78%)	0·01	−4·9% (−10·2 to 0·3)	0·07	−8·5% (−14·0 to −3·0)	0·003
	<1000	321 (87%)	321 (84%)	301 (80%)	0·03	−3·7% (−8·7 to 1·4)	0·15	−7·2% (−12·5 to −1·9)	0·008
Any major or minor PI resistance mutation†		7 (2%)	12 (4%)	32 (11%)	<0·0001	1·4% (−1·2 to 4·0)	0·29	8·6% (4·7 to 12·5)	<0·0001
Viral load <10 000 copies per mL or no major or minor PI resistance mutation/total with viral load		361/367 (98%)	373/383 (97%)	351/375 (94%)	0·001	−1·0% (−3·0 to 1·1)	0·36	−4·8% (−7·6 to 2·0)	0·001
Intermediate/high level LPV/r resistance†		7 (2%)	9 (3%)	31 (11%)	<0·0001	0·5% (−1·9 to 2·9)	0·69	8·3% (4·4 to 12·2)	<0·0001
Intermediate/high level DRV/r resistance†		1 (<1%)	2 (<1%)	12 (3%)	0·001	0·3% (−0·8 to 1·3)	0·59	3·8% (1·4 to 6·1)	0·002
Intermediate/high level NRTI resistance†		10 (3%)	··	··	··	··	··	··	··
Intermediate/high level raltegravir resistance†		··	13 (7%)	··	··	··	··	··	··
Viral load <400 copies per mL									
	Per protocol	306/348 (88%)	296/357 (83%)	··	··	−5·0% (−10·2 to 0·2)	0·06	··	··
	TLOVR	307/426 (72%)	307/433 (71%)	··	··	−0·4% (−3·5 to 2·7) HR 0·97 (0·75 to 1·24)	0·80	··	··
	Snapshot	316/426 (74%)	312/433 (72%)	··	··	−2·1% (−8·1 to 3·8)	0·48	··	··
Good disease control‡		283·6 (67%)	291·9 (67%)	261·8 (63%)	0·31	0·8% (−5·5 to 7·2)	0·80	−3·9% (−10·4 to 2·6)	0·24
Alive		384 (90%)	400 (92%)	387 (93%)	0·37	0·9 (−0·7 to 2·4) HR 1·29 (0·82 to 2·04)	0·27	1·0 (−0·5 to 2·5) HR 1·34 (0·85 to 2·14)	0·21
Alive with no WHO stage 4§		368 (86%)	385 (89%)	371 (89%)	0·74	1·4 (−1·4 to 4·1) HR 1·07 (0·73 to 1·58)	0·73	1·4 (−1·3 to 4·2) HR 1·17 (0·79 to 1·72)	0·44
CD4 count >250 cells per μL		282/366 (77%)	298/385 (77%)	271/377 (72%)	0·15	0·4% (−5·6 to 6·4)	0·91	−5·2% (−11·4 to 1·1)	0·11
CD4 count, mean change		290 (10)	296 (11)	281 (11)	0·61	6 (−23 to 36)	0·66	−8 (−37 to 20)	0·57

Data are n, n (%), n/N (%), or mean (SE), unless otherwise specified. PI=protease inhibitor. NRTI=nucleoside reverse-transcriptase inhibitors. LPV/r=lopinavir plus ritonavir. DRV/r=darunavir plus ritonavir. TLOVR=time to loss of virologic response. HR=hazard ratio. p values given at week 144 from χ^2^ tests for binary endpoints, *t* tests for continuous endpoints, and Cox proportional hazards models for time-to-event endpoints. Any major or minor PI resistance mutation is identically equal to any lopinavir resistance by Stanford (potential low, low, intermediate, or high level). Alive includes the 1·5% lost to follow-up and not known to have died before week 144.

* Absolute risk difference and difference in rate per 100 person-years for binary and time-to-event outcomes (WHO stage 4 or death, death, or TLOVR). HR from Cox proportional hazards model also provided for time-to-event outcomes and is for the good outcome.

† n is number of observed patients with each outcome based on genotype; percentage is of all patients who had viral load measured, using inverse probability weighting within each randomised group to allow for missing genotypes (in 32 [18%] of 182 with viral load >1000 copies per mL); individual NRTI mutations in PI plus NRTI group included zero 65R, eight 70R, nine 67N, six 215Y, six 41L; 37 TAM2, 21 TAM1, and one 151M; raltegravir mutations in PI plus raltegravir group were three 143R, seven 155H, one 148H and 155H, one 97A (minor), and one 66A and 97A (minor); PI mutations most commonly observed were 46I/L (major) in six in PI plus NRTI, five in PI plus raltegravir, and 20 in PI monotherapy; 54V (minor) in four in PI plus NRTI, six in PI plus raltegravir, and 17 in PI monotherapy; 82A/F/S (major) in four in PI plus NRTI, seven in PI plus raltegravir, and 23 in PI monotherapy; 76V (major) in two in PI plus NRTI, four in PI plus raltegravir, and nine in PI monotherapy.

‡ Based on multiple imputation; all other data are as observed (excluding deaths, lost to follow-up, and missed visits).

§ Excluding oesophageal candidiasis and mucosal herpes simplex virus infections.

**Figure 2:**
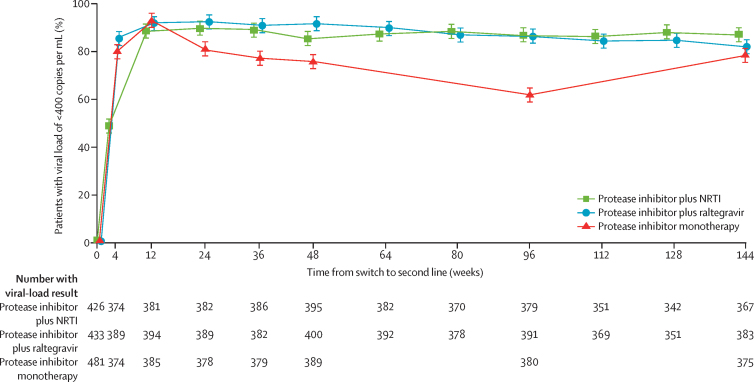
Plasma viral load of less than 400 copies per mL to week 144 in the three treatment groups NRTI=nucleoside reverse-transcriptase inhibitors. GEE=generalised estimating equations. p values comparing the groups by GEE across all weeks from week 36 onwards (testing any direction of effect): global GEE p<0·0001, protease inhibitor plus raltegravir *vs* protease inhibitor plus NRTI GEE p=0·005, protease inhibitor monotherapy *vs* protease inhibitor plus NRTI GEE p<0·0001.

**Figure 3:**
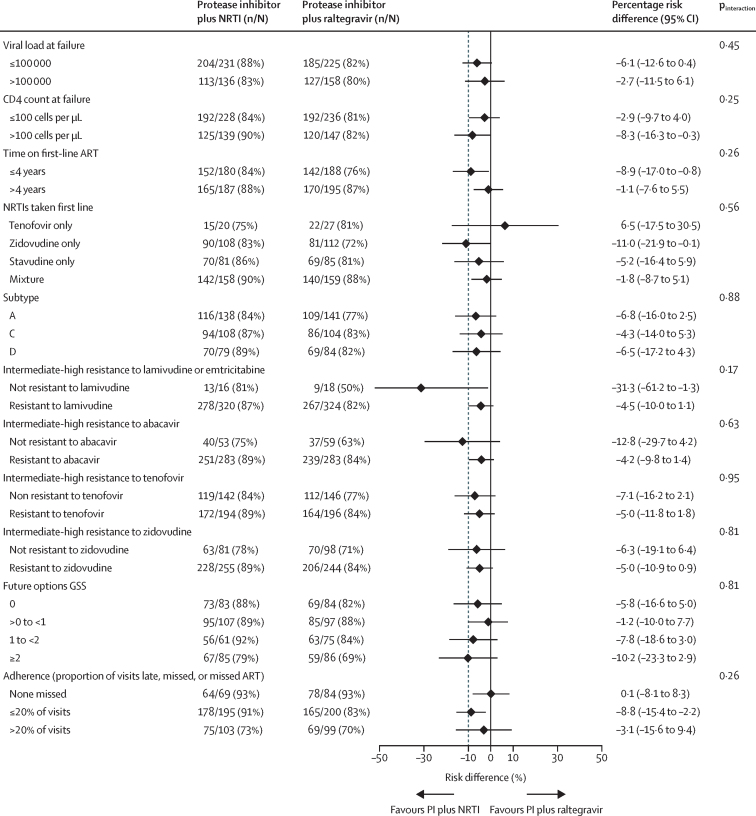
Plasma viral load of less than 400 copies per mL 144 weeks after switch to second-line by analysis approach and key subgroups ART=antiretroviral therapy. GSS=genotypic susceptibility score. NRTI=nucleoside reverse-transcriptase inhibitors. PI=protease inhibitor. For all factors or subgroups tested see appendix (p 3) and for those not shown here see appendix (p 7).

In the protease inhibitor plus raltegravir group, 312 (81%) of 383 participants had viral loads of less than 400 copies per mL, 275 (72%) had viral loads of less than 50 copies per mL, and 321 (84%) had viral loads of less than 1000 copies per mL at week 144 (table 2). Viral-load suppression to less than 400 copies per mL was greater in the protease inhibitor plus raltegravir group than in the protease inhibitor plus NRTI group at week 4 (p<0·0001; figure 2; appendix p 4), but by week 24 there was no evidence of a difference (p=0·19). From week 36 to week 144, a small, but significant, overall difference between the groups initially favoured protease inhibitor plus raltegravir and subsequently protease inhibitor plus NRTI (p=0·005; figure 2). At week 144, for the primary complete-case analysis at less than 400 copies per mL, protease inhibitor plus raltegravir was not superior to protease inhibitor plus NRTI and did not meet the non-inferiority criterion (95% lower confidence limit for the difference exceeded prespecified 10% margin), but was not significantly inferior to protease inhibitor plus NRTI (difference −4·9% [95% CI −10·2 to 0·3]; p=0·07; table 2, appendix p 6). Similar results were seen with the per-protocol analysis (−5·0% [–10·2 to 0·2]; p=0·06; table 2, appendix p 6). Time to loss of virological response (rate difference −0·4% [–3·5 to 2·7]; p= 0·80) and snapshot analyses (difference −2·1% [–8·1 to 3·8]; p=0·48) confirmed that protease inhibitor plus raltegravir was not superior to protease inhibitor plus NRTIs, although the non-inferiority criterion was met (table 2, appendix p 6). Protease inhibitor plus raltegravir did not show superiority in any subgroup and did not meet non-inferiority criteria in many subgroups; there was no evidence that its performance relative to protease inhibitor plus NRTI varied across any subgroup (figure 3, appendix p 7). This pattern of virological outcomes between the protease inhibitor plus raltegravir group and the protease inhibitor plus NRTI group was generally consistent for viral-load suppression at less than 50 copies per mL (difference −3·4% [95% CI −9·7 to 2·9%]; p=0·29) and at less than 1000 copies per mL (−3·7% [–8·7 to 1·4]; p=0·15) on complete-case analyses and across other secondary analyses (appendix pp 6–10). At week 144, intermediate or high-level resistance to raltegravir was seen in 13 participants (7% of protease inhibitor plus raltegravir group overall, adjusting for 29 failed genotypes) and to lopinavir in nine (3%; two with intermediate-level cross-resistance to darunavir; table 2). The proportion of patients with intermediate-high raltegravir resistance in the protease inhibitor plus raltegravir group (7%) did not differ significantly from the proportion with NRTI resistance in the protease inhibitor plus NRTI group (3%; p=0·06).

In the protease inhibitor monotherapy group at week 144, 292 (78%) of 375 participants had viral loads of less than 400 copies per mL, 246 (66%) had viral loads of less than 50 copies per mL and 301 (80%) had viral load of less than 1000 copies per mL (table 2). In protease inhibitor monotherapy, viral-load suppression at less than 400 copies per mL decreased progressively from week 12 (when raltegravir was discontinued) to week 96 (figure 2) and increased at week 144 after the data monitoring committee recommendation to resume combination therapy (difference −8·5% [95% CI −14·0 to −3·0]; p=0·003 *vs* protease inhibitor plus NRTI at week 144). Intermediate or high-level resistance to lopinavir was seen in 31 participants (11%; 12 patients had intermediate-level cross-resistance to darunavir).

There was no difference between the three randomised groups at week 144 in the proportions of patients with good disease control (composite endpoint used for primary week 96 comparison), who were alive, were alive without new WHO stage 4 events, or who had CD4 counts of more than 250 cells per μL (table 2); or in mean CD4 change from baseline (p=0·11 across all timepoints, appendix p 11).

There was no difference between the groups in serious adverse events (global p=0·63), grade 3 or 4 adverse events (total [global p=0·93], or ART-related [global p=0·12]), or events that resulted in treatment modification (global p=0·06; table 3, appendix p 5). In the protease inhibitor plus NRTI group, five patients changed ART because of renal adverse events (two had acute renal failure; both with tenofovir) and five patients changed ART because of haematological adverse events (four had anaemia, one had neutropenia; all with zidovudine). There was no difference between the groups at week 144 in estimated glomerular filtration rate (eGFR) changes from baseline (global p=0·16; appendix p 12), proportions with eGFR of less than 60 ml/min per 1·73 m^2^ (global p=0·83; appendix p 12), haemoglobin changes from baseline (global p=0·73), neurocognitive function changes from baseline (global p=0·89) or proportions with peripheral neuropathy (global p=0·45; table 3).

**Table 3:** Safety outcomes to week 144

			**PI plus NRTI (n=426)**	**PI plus raltegravir (n=433)**	**PI monotherapy (n=418)**	**Global p value**	**PI plus raltegravir *vs* PI plus NRTI**		**PI monotherapy *vs* PI plus NRTI**	
							Risk difference and HR* (95% CI)	p value	Risk difference and HR* (95% CI)	p value
Serious adverse events			113 (27%)	106 (24%)	99 (24%)	0·63	−0·9 (−3·9 to 2·1) HR 0·93 (0·71 to 1·21)	0·57	−1·5 (−4·4 to 1·5) HR 0·88 (0·67 to 1·15)	0·34
	Total number of events		139	127	131	··	··	··	··	··
Grade 3 or 4 events			117 (27%)	118 (27%)	119 (28%)	0·93	0·2 (−2·9 to 3·4) HR 1·02 (0·79 to 1·31)	0·89	0·7 (−2·5 to 3·9) HR 1·05 (0·81 to 1·35)	0·72
	Total grade 3 or 4 events		177	171	161	··	··	··	··	··
		Cardiovascular	3	5	7	··	··	··	··	··
		Respiratory	36	35	32	··	··	··	··	··
		Gastrointestinal	15	13	13	··	··	··	··	··
		Hepatic	4	9	6	··	··	··	··	··
		Renal	9	8	5	··	··	··	··	··
		Central nervous system	19	17	21	··	··	··	··	··
		Skin	6	12	6	··	··	··	··	··
		Haematological	17	12	9	··	··	··	··	··
		Other	68	60	62	··	··	··	··	··
	Grade 3 or 4 events possibly, probably, or definitely ART-related†		26 (6%)	22 (5%)	13 (3%)	0·12	−0·4 (−1·7 to 0·9) HR 0·84 (0·47 to 1·48)	0·54	−1·2 (−2·4 to −0·1) HR 0·50 (0·26 to 0·98)	0·04
		Total events	29	24	14	··	··	··	··	··
Adverse events resulting in ART-modification			25 (6%)	22 (5%)	11 (3%)	0·06	−0·3 (−1·5 to 1·0)	0·66	−1·3 (−2·4 to −2·1)	0·02
Haemoglobin g/dL, mean change‡			1·2 (0·1)	1·2 (0·1)	1·3 (0·1)	0·73	0·0 (−0·3 to 0·3)	0·88	0·1 (−0·2 to 0·4)	0·47
eGFR mL/min per 1·73 m^2^‡										
	Mean change		−12·6 (2·0)	−7·8 (1·8)	−8·8 (1·8)	0·16	4·8 (−0·4 to 10·1)	0·07	3·8 (−1·4 to 9·1)	0·15
	eGFR<60		23/368 (6%)	20/384 (5%)	22/375 (6%)	0·83	−1·0% (−4·4 to 2·3)	0·54	−0·4% (−3·8 to 3·0)	0·83
Neurocognitive *Z* score, mean change‡			1·43 (0·08)	1·42 (0·08)	1·38 (0·08)	0·89	−0·00 (−0·22 to 0·21)	0·98	−0·05 (−0·28 to 0·18)	0·67
Peripheral neuropathy (symptomatic)‡			52/367 (14%)	66/383 (17%)	63/371 (17%)	0·45	3·1% (−2·1 to 8·3)	0·25	2·8 (−2·4 to 8·0)	0·29

Data are n (%), n, mean (SE), or n/N (%), unless otherwise specified. Table shows number of patients who had a particular category of event followed by the total number of events in that category (a patient might have more than one event). PI=protease inhibitor. NRTI=nucleoside reverse-transcriptase inhibitor. ART=antiretroviral therapy. HR=hazard ratio. eGFR=estimated glomerular filtration. p values given at week 144 from χ^2^ tests for binary endpoints, *t* tests for continuous endpoints, and Cox proportional hazards models for time-to-event endpoints. Cockroft-Gault equation was used for eGFR.

* Absolute risk difference for binary outcomes and difference in rate per 100 person-years for time-to-event outcomes (serious adverse events and grade 3 or 4 adverse events). HR from Cox proportional hazards model also provided for time-to-event outcomes.

† Assessed by the clinical expert review committee.

‡ At week 144.

## Discussion

Our findings from this trial, the largest randomised controlled trial of second-line therapy and the only one with 144 weeks' follow-up, show that the robust response we reported previously^3^ in the protease inhibitor plus NRTI group at week 96 is durable, with high rates of viral-load suppression and low rates of drug resistance at 144 weeks. The response was not impaired by the extensive baseline NRTI cross-resistance present in these patients, as discussed elsewhere.^15^ The regimen was also well tolerated. Although most patients who were on protease inhibitor plus NRTI received tenofovir-containing regimens and we used sparse renal toxicity monitoring (achievable in typical programme settings), few patients developed clinically significant renal toxicity. This finding is consistent with reports from similar African programme settings,^16^, ^17^ and shows that intensive renal monitoring is unnecessary in the public health approach in Africa, even in second-line therapy in which serum concentrations of tenofovir are boosted by concomitant protease inhibitor use. More data are needed in Asian patients in whom lower average bodyweight might further increase tenofovir concentrations. A recently licensed pro-drug of tenofovir, tenofovir–alafenamide, has toxicity advantages over the conventional formulation, including lower renal toxicity.^18^ However, based on our data, sparing renal toxicity does not provide a compelling argument for its large-scale adoption in African programmes following the public health approach (although others might exist). Also encouragingly, we found minimal cross-resistance to darunavir after 144 weeks using a lopinavir-based, second-line regimen, which supports the recommended sequence of using lopinavir (combined with NRTIs) for second-line therapy and retaining darunavir for use in third-line therapy, especially as the only trial comparing darunavir with lopinavir in second-line therapy did not show non-inferiority of darunavir.^19^

The original hypothesis underlying this trial was that protease inhibitor plus raltegravir, comprising two new drug classes expected to be fully active, would be superior to protease inhibitor plus NRTI as second-line therapy. However, we found no evidence of virological superiority of protease inhibitor plus raltegravir at 144 weeks in either the primary (complete-case) approach or multiple secondary analyses, consistent with previously reported findings at 48–96 weeks from EARNEST and other trials.^3^, ^4^, ^5^ These earlier reports established non-inferiority of protease inhibitor plus raltegravir, but our findings at week 144 are more equivocal: non-inferiority criteria versus protease inhibitor plus NRTI were not met in the complete-case and per-protocol analysis of viral-load suppression, although the 95% CI lay only marginally outside the specified 10% non-inferiority margin. The protease inhibitor plus raltegravir group did meet non-inferiority criteria on analyses using other viral-load thresholds (50 copies per mL and 1000 copies per mL) and using approaches (TLOVR and snapshot) that counted death as viological failure. This difference between analyses that counted death as virological failure and the complete case and per-protocol analysis, which did not, was primarily because mortality was 2% greater in protease inhibitor plus NRTI, which abrogated the small virological difference favouring protease inhibitor plus NRTI. This small mortality difference between the groups might be a chance finding unrelated to the randomised treatment allocation (it is not statistically significant) and to underlying virological efficacy (the death rate was similar in the protease inhibitor monotherapy group even though virological control was markedly worse). Different analysis approaches sometimes yield contrasting conclusions regarding non-inferiority, but a confident assertion of non-inferiority usually requires consistent results across all analyses: such consistency was not observed.

The explanation for these unexpected findings (absence of virological superiority of protease inhibitor plus raltegravir and inability to demonstrate consistent non-inferiority of this regimen) is unclear, but pharmacokinetic factors might have an important role. Raltegravir has a low genetic barrier to resistance and relatively short half-life and might therefore be more susceptible to the development of resistance during episodes of non-adherence compared with tenofovir, which has a moderate genetic barrier and long intracellular half-life.^20^, ^21^, ^22^ Higher rates of resistance to raltegravir compared with NRTIs observed at week 144 are also consistent with greater fragility of this regimen (although the difference was not statistically significant). Trials of protease inhibitor plus raltegravir combinations in first-line therapy have also shown good virological suppression overall, but less impressive performance in those with high viral load and low CD4 count.^23^, ^24^, ^25^ Although adverse event rates were low with this regimen, we found no evidence of a safety benefit to raltegravir compared with NRTIs in second-line therapy. Dolutegravir, an alternative drug in the integrase strand transfer inhibitor class, which has a higher genetic barrier to resistance and a longer half-life than raltegravir, might be a better candidate for use in combination with a protease inhibitor or NRTIs for second-line therapy in resource-limited settings.^26^, ^27^ 24-week interim data from a trial^28^ comparing a dolutegravir plus NRTI regimen with a standard-of care protease inhibitor plus NRTI regimen in second-line therapy suggest virological superiority of the dolutegravir-containing regimen. However, the trial was done in middle-income countries, with a requirement for resistance testing and selection of patients who had at least one fully active NRTI in the second-line regimen, which limit generalisability to the more challenging situation of low-income countries following the public health approach. Importantly, most participants in EARNEST had no fully active NRTIs in their second-line regimen.^15^ Notably, the restoration of suppression in the protease inhibitor monotherapy group with reintroduction of combination therapy (mainly NRTIs) after week 96 confirms the contribution that NRTIs make to the virological efficacy of a protease inhibitor-based regimen, even when their activity is predicted to be substantially, or even completely, compromised by cross-resistance.

The strengths of this trial are its size, follow-up duration, low withdrawal or loss to follow-up, and regular storage of samples for subsequent centralised viral-load and resistance testing. The broad eligibility criteria and pragmatic approach to delivering treatment (predominantly nurse-led care, clinician drug selection without resistance testing, clinical and immunological efficacy monitoring, sparse laboratory safety monitoring) all enhance generalisability to programme settings in which most patients receive second-line ART, and viral-load testing remains challenging and resistance testing is rarely available.

The main limitation of this study is that treatment was given open-label (which was necessary because it was a pragmatic strategy trial with clinician-selected NRTIs); however, very few patients changed from their allocated treatment strategy and the main outcomes were laboratory parameters assayed blind to treatment received. Most patients were taking a zidovudine or stavudine-based NRTI regimen first-line, and switched to tenofovir at the start of second-line therapy. Current WHO guidelines indicate tenofovir is preferred in first-line, with switch to zidovudine for second-line. However, the virological efficacy of the second-line protease inhibitor plus NRTI regimen does not appear to depend on activity of particular NRTIs, so it is unlikely that this difference in the sequence of NRTIs used would have a substantial impact on our results.^15^

Our findings have important implications for the selection of regimens for second-line therapy in the public health approach. The good longer-term outcomes with the combination of a protease inhibitor (in this case lopinavir) with two NRTIs provides support for this regimen as the WHO-recommended preferred second-line combination.^2^ Dolutegravir might replace efavirenz as standardised first-line therapy in the public health approach in the future, but this should not affect the efficacy of second-line protease inhibitor plus NRTI. Our observations with this regimen are therefore likely to remain relevant for the foreseeable future. Although the protease inhibitor plus raltegravir regimen is currently recommended by WHO as an alternative second-line regimen,^2^ our findings with this combination (absence of virological advantage overall or in any subgroup tested, failure to show non-inferiority consistently across all analyses, absence of a substantive toxicity advantage) taken together with the higher cost of raltegravir, indicate there is no compelling reason for national programmes to adopt this as the standardised second-line therapy.

In settings where therapy can be individualised, a protease inhibitor plus raltegravir regimen could be of value in selected patients if used with regular virological monitoring and adjusted (with drug substitutions) as needed. In the public health approach, a decision to change the standardised sequence of regimens will affect millions of people, and the consequences of an underperforming regimen might not be easily detected and reversed at an individual level. This trial, with its unexpected outcomes, reinforces the need for robust randomised trials with substantial long-term follow-up to be done in resource-limited settings in the populations in whom the public health approach is used before recommendations are changed.
