# Supplementary material for: Lopinavir plus nucleoside reverse-transcriptase inhibitors, lopinavir plus raltegravir, or lopinavir monotherapy for second-line treatment of HIV (EARNEST): 144-week follow-up results from a randomised controlled trial
Source: Lancet Infect Dis. 2018 Jan;18(1):47–57. doi: 10.1016/S1473-3099(17)30630-8 (PMC5739875; doi:10.1016/S1473-3099(17)30630-8)
Supplement: Supplementary appendix [file mmc1.pdf]

THE LANCET Infectious Diseases

Supplementary webappendix

This webappendix formed part of the original submission and has been peer reviewed. We post it as supplied by the authors.

Supplement to: Hakim JG, Thompson J, Kityo C, et al, for the Europe Africa Research Network for Evaluation of Second-line Therapy (EARNEST) Trial Team. Lopinavir plus nucleoside reverse-transcriptase inhibitors, lopinavir plus raltegravir, or lopinavir monotherapy for second-line treatment of HIV (EARNEST): 144-week follow-up results from a randomised controlled trial. *Lancet Infect Dis* 2017; published online Nov 3. http://dx.doi.org/10.1016/S1473-3099(17)30630-8.

SUPPLEMENTARY MATERIALS

Members of the EARNEST Trial Team are:

Participating Sites

Uganda

JCRC Kampala (African trial co-ordinating centre; 231) E Agweng, P Awio, G Bakeinyaga, C Isabirye, U Kabuga, S Kasuswa, M Katuramu, C Kityo, F Kiweewa, H Kyomugisha, E Lutalo, P Mugyenyi, D Mulima, H Musana, G Musitwa, V Musiime, M Ndigendawan, H Namata, J Nkalubo, P Ocitti Labejja, P Okello, P Olal, G Pimundu, P Segonga, F Ssali, Z Tamale, D Tumukunde, W Namala, R Byaruhanga, J Kayiwa, J Tukamushaba, S Abunyang, D Eram, O Denis, R Lwalanda, L Mugarura, J Namusanje, I Nankya, E Ndashimye, E Nabulime, D Mulima, O Senfuma.

IDI, Kampala (216): G Bihabwa, E Buluma, P Easterbrook, A Elbireer, A Kambugu, D Kamya, M Katwere, R Kiggundu, C Komujuni, E Laker, E Lubwama, I Mambule, J Matovu, A Nakajubi, J Nakku, R Nalumenya, L Namuyimbwa, F Semitala, B Wandera, J Wanyama

JCRC, Mbarara (97): H Mugerwa, A Lugemwa, E Ninsiima, T Ssenkindu, S Mwebe, L Atwine, H William, C Katemba, S Abunyang, M Acaku, P Ssebutinde, H Kitizo, J Kukundakwe, M Naluguza, K Ssegawa, Namayanja, F Nsibuka, P Tuhirirwe, M Fortunate

JCRC Fort Portal (66): J Acen, J Achidri, A Amone, M. Chamai, J Ditai, M Kemigisa, M Kiconco, C Matama, D Mbanza, F Nambaziira, M Owor Odoi, A Rweyora, G. Tumwebaze

San Raphael of St Francis Hospital, Nsambya (48): H Kalanzi, J Katabaazi, A Kiyingi, M Mbidde, M. Mugenyi, R Mwebaze, P Okong, I Senoga

JCRC Mbale (47): M Abwola, D Baliruno, J Bwomezi, A Kasede, M Mudoola, R Namisi, F Ssennono, S Tuhirwe

JCRC Gulu (43): G Abongomera, G Amone, J Abach, I Aciro, B Arach, P Kidega, J Omongin, E Ocung, W Odong, A Philliam

JCRC Kabale (33): H Alima, B Ahimbisibwe, E Atuhaire, F Atukunda, G Bekusike, A Bulegyeya, D. Kahatano, S Kamukama, J Kyoshabire, A Nassali, A Mbonye, T M Naturinda, Ndukukire, A Nshabohurira, H. Ntawiha, A Rogers, M Tibyasa;

JCRC Kakira (31): S. Kiirya, D. Atwongyeire, A. Nankya, C. Draleku, D. Nakiboneka, D. Odoch, L. Lakidi, R. Ruganda, R. Abiriga, M. Mulindwa, F. Balmoi, S. Kafuma, E. Moriku

Zimbabwe

University of Zimbabwe Clinical Research Centre, Harare (265): J Hakim, A Reid, E Chidziva, G Musoro, C Warambwa, G Tinago, S Mutsai, M Phiri, S Mudzingwa, T Bafana, V Masore, C Moyo, R Nhema, S Chitongo

Malawi

Department of Medicine, University of Malawi College of Medicine and the Malawi-Liverpool-Wellcome Trust Clinical Research Programme, University of Malawi College of Medicine (92): Robert Heyderman, Lucky Kabanga, Symon Kaunda, Aubrey Kudzala, Linly Lifa, Jane Mallewa, Mike Moore, Chrissie Mtali, George Musowa, Grace Mwimaniwa, Rosemary Sikwese, Joep van Oosterhout, Milton Ziwoya

Mzuzu Central Hospital, Mzuzu (19): H Chimbaka. B Chitete, S Kamanga, T Kayinga E Makwakwa, R Mbiya, M Mlenga, T Mphande, C Mtika, G Mushani, O Ndhlovu, M Ngonga, I Nkhana, R Nyirenda

Kenya

Moi Teaching and Referral Hospital (52): P Cheruiyot, C Kwobah, W Lokitala Ekiru, M Mokaya, A Mudogo, A Nzioka, A Siika, M Tanui, S Wachira, K Wools-Kaloustian

Zambia

University Teaching Hospital (37): P Alipalli, E Chikatula, J Kipaila, I Kunda, S Lakhi, J Malama, W Mufwambi, L Mulenga, P Mwaba, E Mwamba, A Mweemba, M Namfukwe

The Aids Support Organisation (TASO), Uganda: E Kerukadho, B Ngwatu, J Birungi

MRC Clinical Trials Unit: N Paton, J Boles, A Burke, L Castle, S Ghuman, L Kendall, A Hoppe, S Tebbs, M Thomason, J Thompson, AS Walker, J Whittle, H Wilkes, N Young, M Spyer

Monitors: C Kapuya, F Kyomuhendo, D Kyakundi, N Mkandawire, S Mulambo, S Senyonjo

Clinical Expert Review Committee: B Angus, A Arenas-Pinto, A Palfreeman, F Post, D Ishola

European Collaborators:

J Arribas (Hospital La Paz, Madrid, Spain), R Colebunders (Institute of Tropical Medicine, Antwerp, Belgium), M Floridia (ISS, Italy), M Giuliano (ISS, Italy), P Mallon (University College Dublin, Ireland), P Walsh (University College Dublin, Ireland), M De Rosa (CINECA, Italy), E Rinaldi (CINECA, Italy)

Trial Steering Committee: I Weller (Chair), C Gilks, J Hakim, A Kangewende, S Lakhi, E Luyirika, F Miiro, P Mwamba, P Mugyenyi, S Ojoo, N Paton, S Phiri, J van Oosterhout, A Siika, S Walker, A Wapakabulo,

Data Monitoring Committee: T Peto (Chair), N French, J Matenga

Pharmaceutical companies: G Cloherty, J van Wyk, M Norton, S Lehrman, P Lamba, K Malik, J Rooney, W Snowden, J Villacian

SUPPLEMENTARY METHODS

Factors/subgroups tested were VL and CD4 count at switch, duration of first-line ART, NRTIs taken during first-line and at failure, and NRTIs specified by physicians prior to randomisation that they would prescribe if the patients were randomised to PI/NRTI (available for both groups, in contrast to actual NRTIs prescribed which is only available for PI/NRTI), viral subtype (based on available genotypes), presence of intermediate-high resistance to NRTIs, number of NRTIs available with susceptibility/low-level resistance, presence of M184V mutation, a future options genotypic susceptibility score (GSS; obtained from the total of the scores for predicted susceptibility of 3TC/FTC, ABC, D4T, DDI, TDF and ZDV determined using the Stanford algorithm according to predicted susceptibility), adherence to second-line (percentage of visits reporting missing pills in the last month, missed, or attended over 7 days late) and self-reported alcohol consumption during second-line, and employment status. VL, CD4, and duration of first-line ART were subgroups specified in the Statistical Analysis plan; other factors were exploratory.

SUPPLEMENTARY TABLES

Supplementary Table 1: Early virological responses on second-line therapy by randomized group

	PI/NRTI	PI/RAL	Difference PI/RAL-PI/NRTI (95% CI)	P value
**Week 4**				
Available, N	374	389		
VL<50 copies/ml, N (%)	31 (8%)	125 (32%)	+23.8% (+18.4%, +29.3%)	<0.0001
VL<400 copies/ml, N (%)	180 (48%)	333 (86%)	+37.5% (+31.3%, +43.6%)	<0.0001
VL<1000 copies/ml, N (%)	278 (74%)	360 (93%)	+18.2% (+13.1%, +23.4%)	<0.0001
Change in $\log_{10}$ VL from baseline, mean (95% CI)*	-2.13 (-2.22, -2.03)	-2.88 (-2.97, -2.78)	-0.75 (-0.88, -0.62)	<0.0001
**Week 12**				
Available, N	381	394		
VL<50 copies/ml, N (%)	163 (43%)	259 (66%)	+23.0% (+16.1%, +29.8%)	<0.0001
VL<400 copies/ml, N (%)	335 (88%)	361 (92%)	+3.7% (-0.6%, +8.0%)	0.09
VL<1000 copies/ml, N (%)	357 (94%)	367 (93%)	-0.6% (-4.0%, +2.9%)	0.76
**Week 24**				
Available, N	382	389		
VL<50 copies/ml, N (%)	266 (70%)	299 (77%)	+7.2% (+1.0%, +13.5%)	0.02
VL<400 copies/ml, N (%)	341 (89%)	358 (92%)	+2.8% (-1.3%, +6.9%)	0.19
VL<1000 copies/ml, N (%)	350 (92%)	363 (93%)	+1.7% (-2.0%, +5.4%)	0.37

*Declines in $\log_{10}$VL from baseline to week 4 and 12 were compared between the PI/NRTI and PI/RAL groups (PI-mono same as PI/RAL to week 12) using interval regression (left censoring those <40 copies/ml at week 4 at 0 $\log_{10}$ copies/ml; similar results censoring at 39 $\log_{10}$ 39 copies/ml, not shown).

Supplementary Table 2: Serious Adverse Events to week 144, by category

	PI/NRTI (N=426)	PI/RAL (N=433)	PI-mono (N=418)
SAE – n patients (%)	113 (27%)	106 (24%)	99 (24%)
Total SAEs	139	127	131
Fatal	42	33	31
Life threatening	18	18	13
Caused or prolonged hospitalisation	98	89	92
Persistent or significant disability/incapacity	1	3	1
Congenital anomaly/ birth defect	0	0	0
Other important medical condition	3	7	11

Table shows number of patients experiencing an SAE (n, %) followed by the total number of SAEs (a patient may have more than one SAE)

SUPPLEMENTARY FIGURES

Supplementary Figure 1: Plasma VL<400 copies/ml 144 weeks after switch to second-line in PI/NRTI vs PI/RAL by analysis approach

	PI/NRTI	PI/RAL	Percentage Risk Difference/ Rate Difference (95% CI)	P-value
VL<50				
ITT	276/367(75%)	275/383(72%)	-3.4 (-9.7, 2.9)	0.29
PER PROTOCOL	270/348(78%)	260/357(73%)	-4.8 (-11.1, 1.6)	0.14
SNAPSHOT	276/426(65%)	275/433(64%)	-1.3 (-7.7, 5.1)	0.70
TLOVR	223/426(52%)	228/433(53%)	1.0 (-3.8, 5.9)	0.67
VL<400				
ITT	317/367(86%)	312/383(81%)	-4.9 (-10.2, 0.3)	0.07
PER PROTOCOL	306/348(88%)	296/357(83%)	-5.0 (-10.2, 0.2)	0.06
SNAPSHOT	316/426(74%)	312/433(72%)	-2.1 (-8.1, 3.8)	0.48
TLOVR	307/426(72%)	307/433(71%)	-0.4 (-3.5, 2.7)	0.80
VL<1000				
ITT	321/367(87%)	321/383(84%)	-3.7 (-8.7, 1.4)	0.15
PER PROTOCOL	309/348(89%)	305/357(85%)	-3.4 (-8.3, 1.6)	0.18
SNAPSHOT	320/426(75%)	321/433(74%)	-1.0 (-6.8, 4.8)	0.74
TLOVR	312/426(73%)	316/433(73%)	0.0 (-2.9, 3.0)	0.98

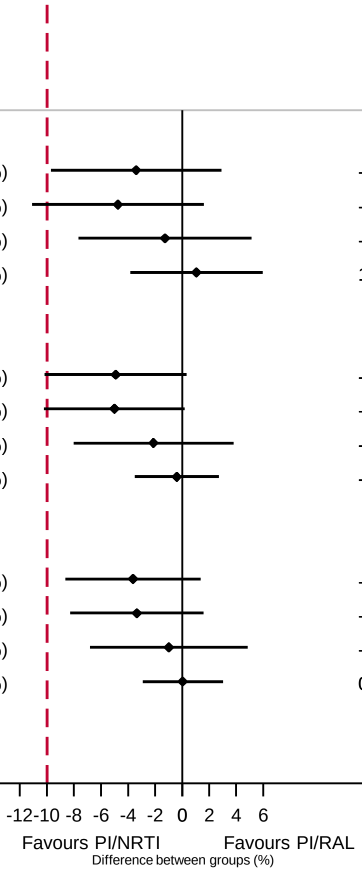

TLOVR result given as absolute rate difference per 100 person years

Supplementary figure 2: Plasma VL<400 copies/ml 144 weeks after switch to second-line in PI/NRTI vs PI/RAL by all subgroups tested

	PI/NRTI	PI/RAL	Percentage Risk difference (95% CI)	Interaction p-value
Viral Load at failure				
≤ 100,000	204/231(88%)	185/225(82%)	-6.1 (-12.6, 0.4)	0.45
>100,000	113/136(83%)	127/158(80%)	-2.7 (-11.5, 6.1)	
CD4 at failure				
≤ 100	192/228(84%)	192/236(81%)	-2.9 (-9.7, 4.0)	0.25
>100	125/139(90%)	120/147(82%)	-8.3 (-16.3, -0.3)	
Time on first line ART				
≤ 4 years	152/180(84%)	142/188(76%)	-8.9 (-17.0, -0.8)	0.26
> 4years	165/187(88%)	170/195(87%)	-1.1 (-7.6, 5.5)	
NRTIs taken first line				
TDF only	15/20(75%)	22/27(81%)	6.5 (-17.5, 30.5)	0.56
ZDV only	90/108(83%)	81/112(72%)	-11.0 (-21.9, -0.1)	
D4T only	70/81(86%)	69/85(81%)	-5.2 (-16.4, 5.9)	
Mixture	142/158(90%)	140/159(88%)	-1.8 (-8.7, 5.1)	
On D4T at failure				
Not taking d4t at failure	241/280(86%)	238/293(81%)	-4.8 (-10.9, 1.2)	0.93
Taking d4t at failure	76/87(87%)	74/90(82%)	-5.1 (-15.7, 5.4)	
On TDF at failure				
Not taking tdf at failure	288/331(87%)	260/322(81%)	-6.3 (-11.9, -0.6)	0.18
Taking tdf at failure	29/36(81%)	52/61(85%)	4.7 (-11.0, 20.4)	
On ZDV at failure				
Not taking zdv at failure	108/127(85%)	128/152(84%)	-0.8 (-9.3, 7.7)	0.25
Taking zdv at failure	209/240(87%)	184/231(80%)	-7.4 (-14.1, -0.7)	
NRTIs would have received if randomised to PI + NRTI				
3TC/TDF if randomised to NRTIs	228/262(87%)	218/272(80%)	-6.9 (-13.1, -0.6)	0.77
ABC/DDI if randomised to NRTIs	38/40(95%)	44/49(90%)	-5.2 (-16.0, 5.6)	
Subtype				
Subtype A	116/138(84%)	109/141(77%)	-6.8 (-16.0, 2.5)	0.88
Subtype C	94/108(87%)	86/104(83%)	-4.3 (-14.0, 5.3)	
Subtype D	70/79(89%)	69/84(82%)	-6.5 (-17.2, 4.3)	
Intermediate-high resistance to 3TC/FTC				
Not resistant to 3TC	13/16(81%)	9/18(50%)	-31.3 (-61.2, -1.3)	0.17
Resistant to 3TC	278/320(87%)	267/324(82%)	-4.5 (-10.0, 1.1)	
Intermediate-high resistance to ABC				
Not resistant to ABC	40/53(75%)	37/59(63%)	-12.8 (-29.7, 4.2)	0.63
Resistant to ABC	251/283(89%)	239/283(84%)	-4.2 (-9.8, 1.4)	
Intermediate-high resistance to TDF				
Not resistant to TDF	119/142(84%)	112/146(77%)	-7.1 (-16.2, 2.1)	0.95
Resistant to TDF	172/194(89%)	164/196(84%)	-5.0 (-11.8, 1.8)	
Intermediate-high resistance to ZDV				
Not resistant to ZDV	63/81(78%)	70/98(71%)	-6.3 (-19.1, 6.4)	0.81
Resistant to ZDV	228/255(89%)	206/244(84%)	-5.0 (-10.9, 0.9)	
N susceptible-low resistance in ABC, DDI, TDF, ZDV				
0 active	158/178(89%)	142/170(84%)	-5.2 (-12.5, 2.0)	0.94
1 active	64/71(90%)	72/83(87%)	-3.4 (-13.5, 6.7)	
2 active	30/35(86%)	26/31(84%)	-1.8 (-19.2, 15.5)	
4 active	39/52(75%)	36/58(62%)	-12.9 (-30.1, 4.2)	
Presence of M184V				
No mutation	23/26(88%)	18/28(64%)	-24.2 (-45.8, -2.6)	0.14
Mutation present	268/310(86%)	258/314(82%)	-4.3 (-10.0, 1.4)	
Future Options GSS				
0	73/83(88%)	69/84(82%)	-5.8 (-16.6, 5.0)	0.81
>0 - <1	95/107(89%)	85/97(88%)	-1.2 (-10.0, 7.7)	
1 - <2	56/61(92%)	63/75(84%)	-7.8 (-18.6, 3.0)	
2+	67/85(79%)	59/86(69%)	-10.2 (-23.3, 2.9)	
Adherence (proportion of visits late, missed, or missed ART)				
None missed	64/69(93%)	78/84(93%)	0.1 (-8.1, 8.3)	0.26
≤ 20% of visits	178/195(91%)	165/200(83%)	-8.8 (-15.4, -2.2)	
>20% of visits	75/103(73%)	69/99(70%)	-3.1 (-15.6, 9.4)	
Alcohol consumption				
Never drinks	250/285(88%)	250/303(83%)	-5.2 (-10.9, 0.5)	0.88
Ever drinks	56/66(85%)	41/53(77%)	-7.5 (-21.7, 6.7)	
Employment at failure				
Unemployed	83/101(82%)	90/106(85%)	2.7 (-7.4, 12.8)	0.06
Employed	234/265(88%)	220/275(80%)	-8.3 (-14.4, -2.2)	

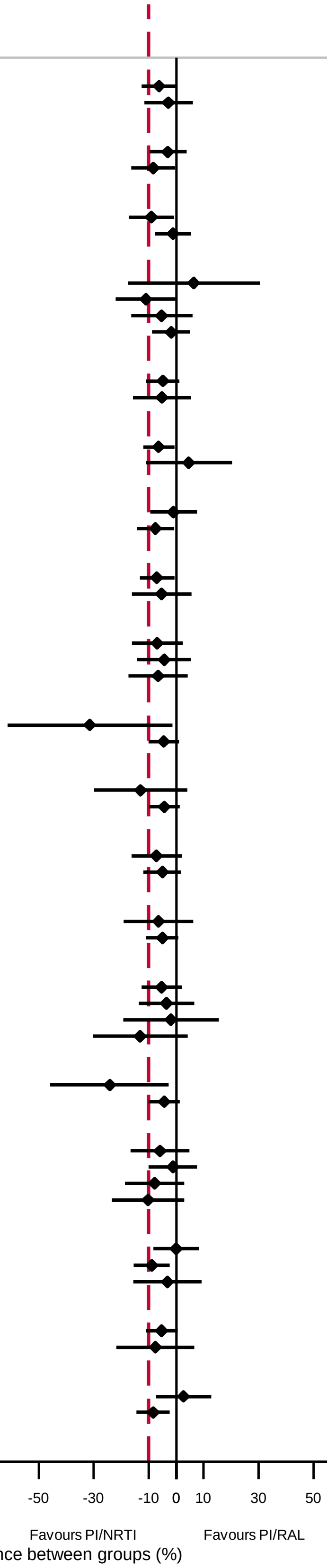

Note: See Supplementary Methods for details on factors tested.

Supplementary Fig 3a: Plasma VL<50 copies/ml to week 144 in the three second-line treatment groups

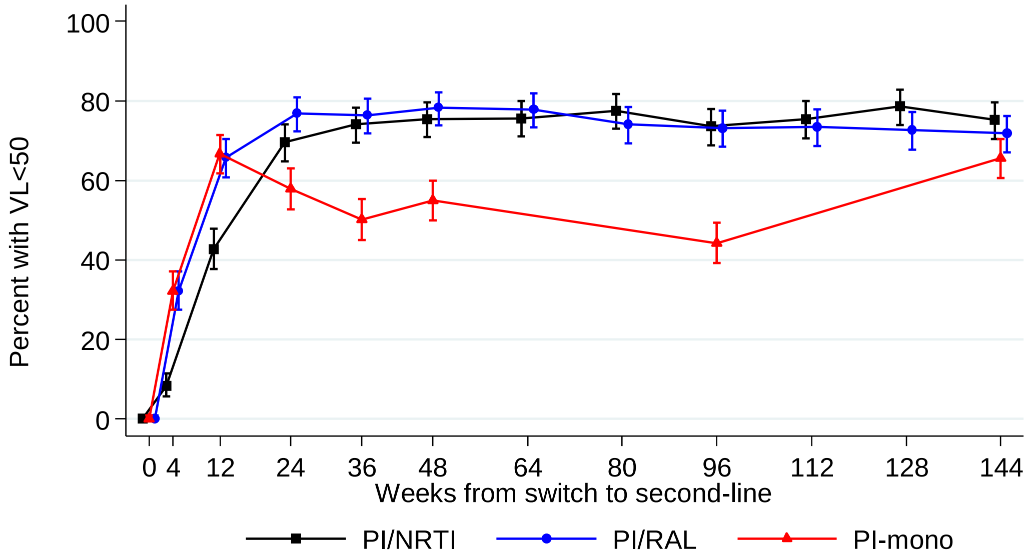

P values comparing the groups by Generalised Estimating Equations across all weeks from week 36 onwards (testing any direction of effect):
Global GEE p<0.0001
PI/RAL vs PI/NRTI GEE p=0.19
PI-mono vs PI/NRTI GEE p<0.0001

Supplementary figure 3b: Plasma VL<1000 copies/ml to week 144 in the three second-line treatment groups

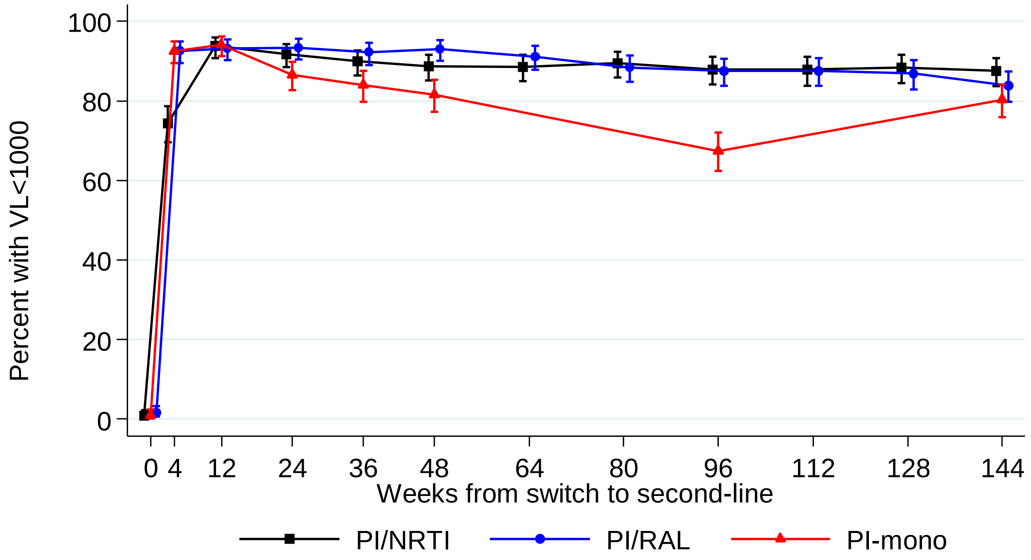

P values comparing the groups by Generalised Estimating Equations across all weeks from week 36 onwards (testing any direction of effect):
Global GEE p<0.0001
PI/RAL vs PI/NRTI GEE p=0.07
PI-mono vs PI/NRTI GEE p<0.0001

Supplementary figure 4: Plasma VL<50 copies/ml 144 weeks after switch to second-line in PI/NRTI vs PI/RAL by all subgroups tested

	PI/NRTI	PI/RAL	Percentage Risk difference (95% CI)	Interaction p-value
Viral Load at failure				
≤100,000	183/231(79%)	166/225(74%)	-5.4 (-13.2, 2.3)	0.32
>100,000	93/136(68%)	109/158(69%)	0.6 (-10.0, 11.2)	
CD4 at failure				
≤ 100	168/228(74%)	170/236(72%)	-1.7 (-9.7, 6.4)	0.47
>100	108/139(78%)	105/147(71%)	-6.3 (-16.3, 3.8)	
Time on first line ART				
≤ 4 years	132/180(73%)	125/188(66%)	-6.8 (-16.2, 2.5)	0.33
> 4years	144/187(77%)	150/195(77%)	-0.1 (-8.5, 8.4)	
NRTIs taken first line				
TDF only	13/20(65%)	17/27(63%)	-2.0 (-29.8, 25.7)	0.89
ZDV only	80/108(74%)	75/112(67%)	-7.1 (-19.1, 4.9)	
D4T only	61/81(75%)	61/85(72%)	-3.5 (-17.0, 9.9)	
Mixture	122/158(77%)	122/159(77%)	-0.5 (-9.8, 8.8)	
On D4T at failure				
Not taking d4t at failure	210/280(75%)	209/293(71%)	-3.7 (-10.9, 3.6)	0.89
Taking d4t at failure	66/87(76%)	66/90(73%)	-2.5 (-15.3, 10.3)	
On TDF at failure				
Not taking tdf at failure	253/331(76%)	233/322(72%)	-4.1 (-10.8, 2.6)	0.36
Taking tdf at failure	23/36(64%)	42/61(69%)	5.0 (-14.6, 24.5)	
On ZDV at failure				
Not taking zdv at failure	92/127(72%)	109/152(72%)	-0.7 (-11.3, 9.8)	0.53
Taking zdv at failure	184/240(77%)	166/231(72%)	-4.8 (-12.7, 3.1)	
NRTIs would have received if randomised to PI + NRTI				
3TC/TDF if randomised to NRTIs	196/262(75%)	194/272(71%)	-3.5 (-11.0, 4.0)	0.83
ABC/DDI if randomised to NRTIs	35/40(88%)	41/49(84%)	-3.8 (-18.4, 10.7)	
Subtype				
Subtype A	100/138(72%)	93/141(66%)	-6.5 (-17.3, 4.3)	0.25
Subtype C	82/108(76%)	78/104(75%)	-0.9 (-12.5, 10.7)	
Subtype D	63/79(80%)	61/84(73%)	-7.1 (-20.1, 5.9)	
Intermediate-high resistance to 3TC/FTC				
Not resistant to 3TC	12/16(75%)	9/18(50%)	-25.0 (-56.4, 6.4)	0.19
Resistant to 3TC	240/320(75%)	237/324(73%)	-1.9 (-8.6, 4.9)	
Intermediate-high resistance to ABC				
Not resistant to ABC	34/53(64%)	35/59(59%)	-4.8 (-22.8, 13.2)	0.87
Resistant to ABC	218/283(77%)	211/283(75%)	-2.5 (-9.5, 4.6)	
Intermediate-high resistance to TDF				
Not resistant to TDF	103/142(73%)	101/146(69%)	-3.4 (-13.8, 7.1)	0.98
Resistant to TDF	149/194(77%)	145/196(74%)	-2.8 (-11.4, 5.7)	
Intermediate-high resistance to ZDV				
Not resistant to ZDV	57/81(70%)	62/98(63%)	-7.1 (-20.9, 6.7)	0.49
Resistant to ZDV	195/255(76%)	184/244(75%)	-1.1 (-8.6, 6.4)	
N susceptible-low resistance in ABC, DDI, TDF, ZDV				
0 active	135/178(76%)	127/170(75%)	-1.1 (-10.2, 7.9)	0.90
1 active	58/71(82%)	64/83(77%)	-4.6 (-17.3, 8.2)	
2 active	26/35(74%)	20/31(65%)	-9.8 (-32.0, 12.4)	
4 active	33/52(63%)	35/58(60%)	-3.1 (-21.3, 15.0)	
Presence of M184V				
No mutation	21/26(81%)	17/28(61%)	-20.1 (-43.7, 3.5)	0.16
Mutation present	231/310(75%)	229/314(73%)	-1.6 (-8.5, 5.3)	
Future Options GSS				
0	61/83(73%)	59/84(70%)	-3.3 (-16.9, 10.4)	0.13
>0 - <1	81/107(76%)	80/97(82%)	6.8 (-4.3, 17.9)	
1 - <2	53/61(87%)	54/75(72%)	-14.9 (-28.1, -1.7)	
2+	57/85(67%)	53/86(62%)	-5.4 (-19.8, 8.9)	
Adherence (proportion of visits late, missed, or missed ART)				
None missed	55/69(80%)	73/84(87%)	7.2 (-4.7, 19.1)	0.11
≤ 20% of visits	158/195(81%)	144/200(72%)	-9.0 (-17.3, -0.7)	
>20% of visits	63/103(61%)	58/99(59%)	-2.6 (-16.1, 10.9)	
Alcohol consumption				
Never drinks	215/285(75%)	224/303(74%)	-1.5 (-8.5, 5.5)	0.07
Ever drinks	52/66(79%)	32/53(60%)	-18.4 (-34.9, -2.0)	
Employment at failure				
Unemployed	69/101(68%)	81/106(76%)	8.1 (-4.1, 20.2)	0.02
Employed	207/265(78%)	192/275(70%)	-8.3 (-15.7, -0.9)	

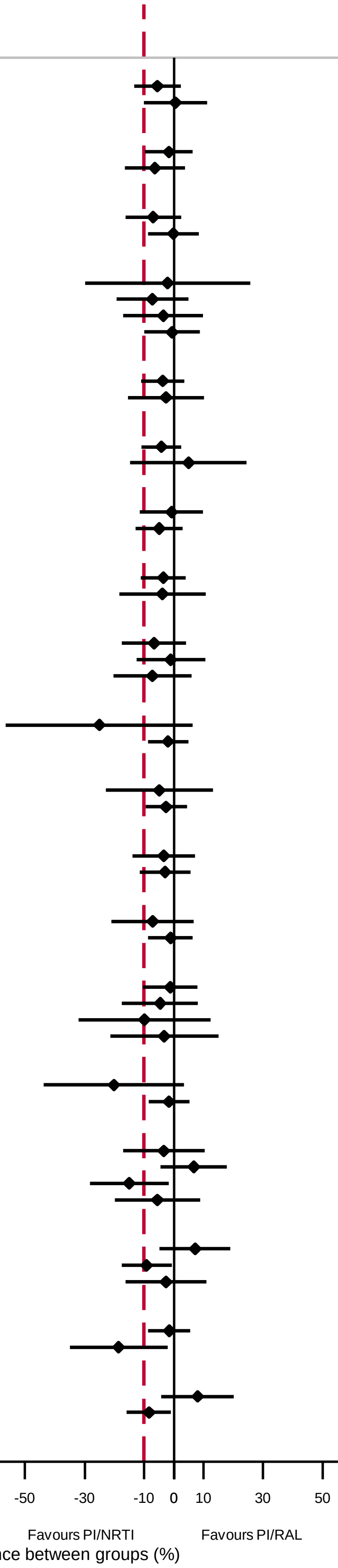

Note: See Supplementary Methods for information on factors tested. 19 subgroup analyses performed, and therefore ~1 interaction p-value would be expected to be <0.05 by chance

Supplementary figure 5: Plasma VL<1000 copies/ml at 144 weeks after switch to second-line in PI/NRTI vs PI/RAL by all subgroups tested

	PI/NRTI	PI/RAL	Percentage Risk difference (95% CI)	Interaction p-value
Viral Load at failure				
≤100,000	205/231(89%)	190/225(84%)	-4.3 (-10.5, 1.9)	0.65
>100,000	116/136(85%)	131/158(83%)	-2.4 (-10.7, 6.0)	
CD4 at failure				
≤ 100	196/228(86%)	198/236(84%)	-2.1 (-8.6, 4.4)	0.38
>100	125/139(90%)	123/147(84%)	-6.3 (-14.0, 1.5)	
Time on first line ART				
≤ 4 years	154/180(86%)	148/188(79%)	-6.8 (-14.6, 1.0)	0.34
> 4years	167/187(89%)	173/195(89%)	-0.6 (-6.9, 5.7)	
NRTIs taken first line				
TDF only	15/20(75%)	23/27(85%)	10.2 (-13.0, 33.4)	0.44
ZDV only	92/108(85%)	86/112(77%)	-8.4 (-18.7, 1.9)	
D4T only	71/81(88%)	69/85(81%)	-6.5 (-17.4, 4.5)	
Mixture	143/158(91%)	143/159(90%)	-0.6 (-7.1, 6.0)	
On D4T at failure				
Not taking d4t at failure	244/280(87%)	247/293(84%)	-2.8 (-8.6, 2.9)	0.58
Taking d4t at failure	77/87(89%)	74/90(82%)	-6.3 (-16.6, 4.1)	
On TDF at failure				
Not taking tdf at failure	292/331(88%)	267/322(83%)	-5.3 (-10.7, 0.1)	0.09
Taking tdf at failure	29/36(81%)	54/61(89%)	8.0 (-7.2, 23.2)	
On ZDV at failure				
Not taking zdv at failure	109/127(86%)	130/152(86%)	-0.3 (-8.6, 8.0)	0.31
Taking zdv at failure	212/240(88%)	191/231(83%)	-5.6 (-12.0, 0.7)	
NRTIs would have received if randomised to PI + NRTI				
3TC/TDF if randomised to NRTIs	231/262(88%)	226/272(83%)	-5.1 (-11.0, 0.8)	0.35
ABC/DDI if randomised to NRTIs	39/40(98%)	44/49(90%)	-7.7 (-17.5, 2.1)	
Subtype				
Subtype A	119/138(86%)	115/141(82%)	-4.7 (-13.3, 3.9)	0.78
Subtype C	95/108(88%)	86/104(83%)	-5.3 (-14.8, 4.2)	
Subtype D	70/79(89%)	70/84(83%)	-5.3 (-15.9, 5.3)	
Intermediate-high resistance to 3TC/FTC				
Not resistant to 3TC	13/16(81%)	9/18(50%)	-31.3 (-61.2, -1.3)	0.15
Resistant to 3TC	282/320(88%)	275/324(85%)	-3.2 (-8.5, 2.0)	
Intermediate-high resistance to ABC				
Not resistant to ABC	40/53(75%)	39/59(66%)	-9.4 (-26.1, 7.4)	0.82
Resistant to ABC	255/283(90%)	245/283(87%)	-3.5 (-8.8, 1.7)	
Intermediate-high resistance to TDF				
Not resistant to TDF	119/142(84%)	115/146(79%)	-5.0 (-14.0, 3.9)	0.80
Resistant to TDF	176/194(91%)	169/196(86%)	-4.5 (-10.8, 1.8)	
Intermediate-high resistance to ZDV				
Not resistant to ZDV	63/81(78%)	73/98(74%)	-3.3 (-15.8, 9.2)	0.55
Resistant to ZDV	232/255(91%)	211/244(86%)	-4.5 (-10.1, 1.0)	
N susceptible-low resistance in ABC, DDI, TDF, ZDV				
0 active	162/178(91%)	146/170(86%)	-5.1 (-11.8, 1.6)	0.86
1 active	64/71(90%)	73/83(88%)	-2.2 (-12.0, 7.7)	
2 active	30/35(86%)	27/31(87%)	1.4 (-15.2, 17.9)	
4 active	39/52(75%)	38/58(66%)	-9.5 (-26.5, 7.5)	
Presence of M184V				
No mutation	23/26(88%)	18/28(64%)	-24.2 (-45.8, -2.6)	0.12
Mutation present	272/310(88%)	266/314(85%)	-3.0 (-8.4, 2.4)	
Future Options GSS				
0	76/83(92%)	70/84(83%)	-8.2 (-18.2, 1.7)	0.58
>0 - <1	96/107(90%)	88/97(91%)	1.0 (-7.1, 9.2)	
1 - <2	56/61(92%)	64/75(85%)	-6.5 (-17.0, 4.1)	
2+	67/85(79%)	62/86(72%)	-6.7 (-19.6, 6.1)	
Adherence (proportion of visits late, missed, or missed ART)				
None missed	65/69(94%)	79/84(94%)	-0.2 (-7.6, 7.3)	0.40
≤ 20% of visits	179/195(92%)	170/200(85%)	-6.8 (-13.1, -0.5)	
>20% of visits	77/103(75%)	72/99(73%)	-2.0 (-14.2, 10.1)	
Alcohol consumption				
Never drinks	251/285(88%)	255/303(84%)	-3.9 (-9.5, 1.7)	0.58
Ever drinks	58/66(88%)	42/53(79%)	-8.6 (-22.1, 4.8)	
Employment at failure				
Unemployed	85/101(84%)	95/106(90%)	5.5 (-3.7, 14.7)	0.02
Employed	236/265(89%)	224/275(81%)	-7.6 (-13.5, -1.7)	

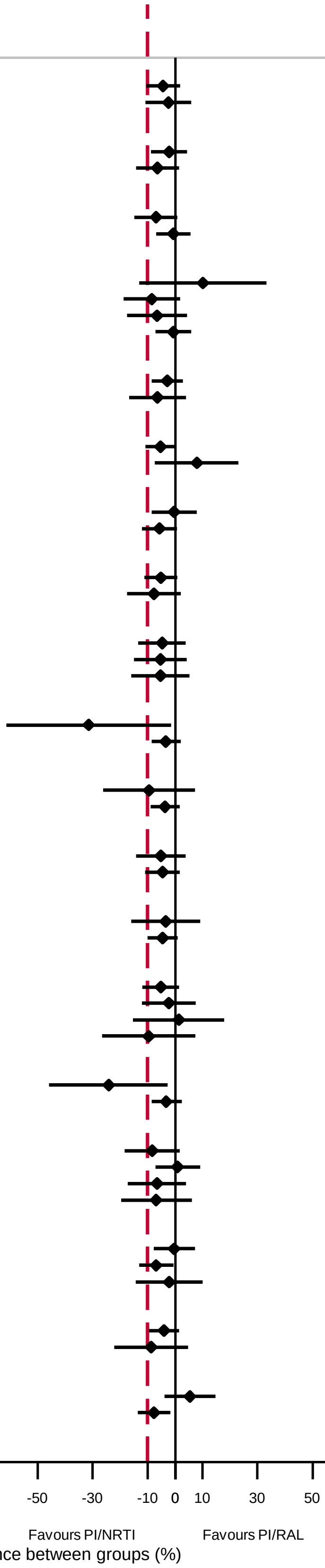

Note: See footnote to Figure 2 for information on factors tested. 19 subgroup analyses performed, and therefore ~1 interaction p-value would be expected to be <0.05 by chance

Supplementary figure 6: Change in CD4 count to week 144 by treatment group

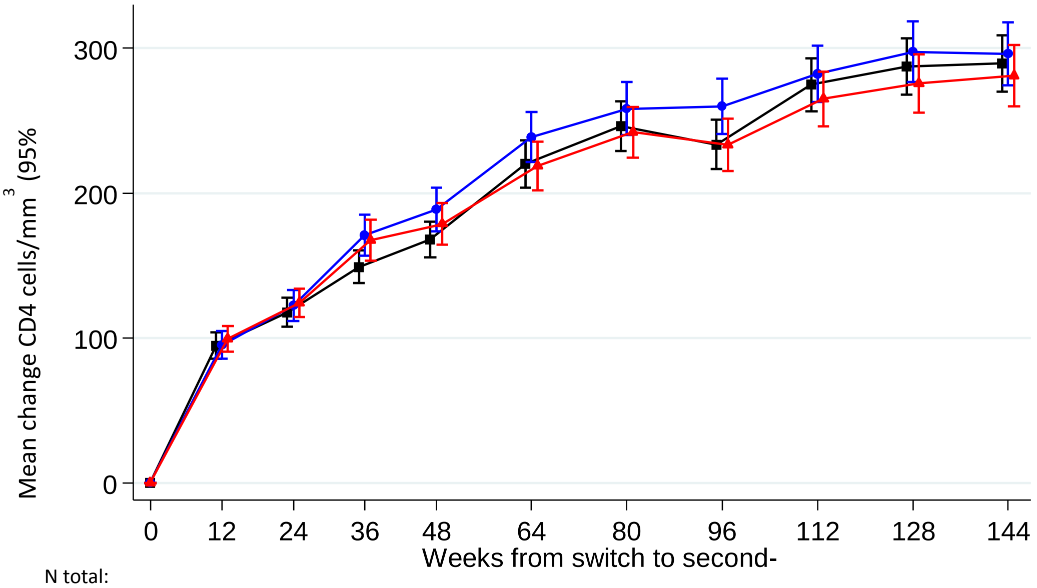

N total:

PI/NRTI	426	401	402	386	389	386	380	381	371	367	366
PI/RAL	433	410	402	391	495	391	387	390	384	380	385
PI-mono	418	401	391	376	393	385	376	379	374	367	377

PI/NRTI PI/RAL PI-

P values comparing the groups by Generalised Estimating Equations across all weeks (testing any direction of effect):
Global GEE p=0.11
PI/RAL vs PI/NRTI GEE p=0.04
PI-mono vs PI/NRTI GEE p=0.30

Supplementary figure 7a: Change in eGFR to week 144 by treatment group

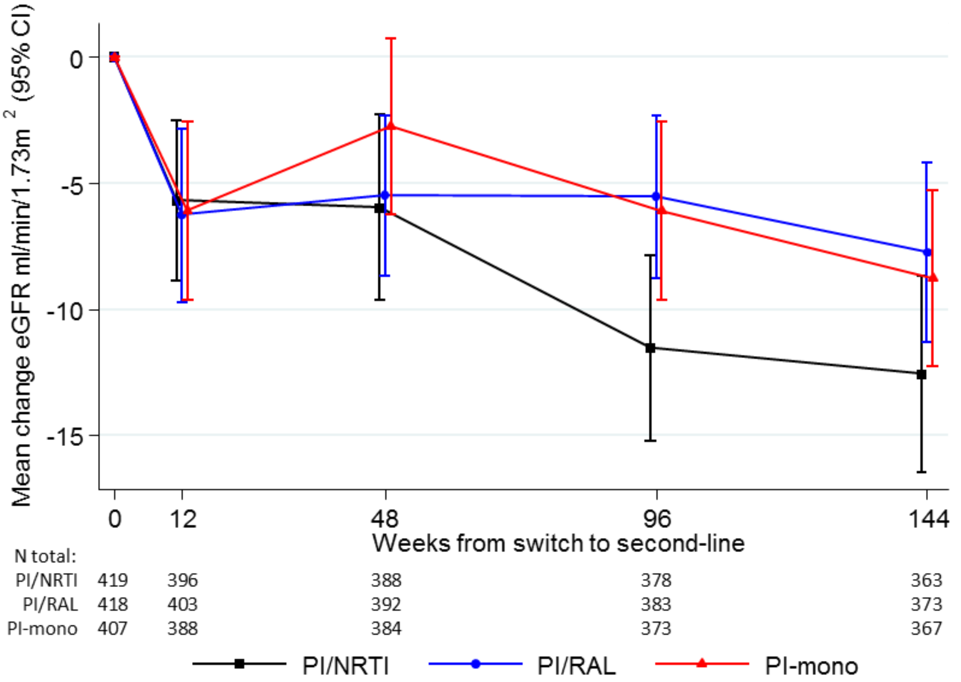

P values comparing the groups by Generalised Estimating Equations across all weeks (testing any direction of effect):
Global GEE p=0.06
PI/RAL vs PI/NRTI GEE p=0.02
PI-mono vs PI/NRTI GEE p=0.21

Supplementary figure 7b: Proportion with grade 2 or higher eGFR to week 144 by treatment group

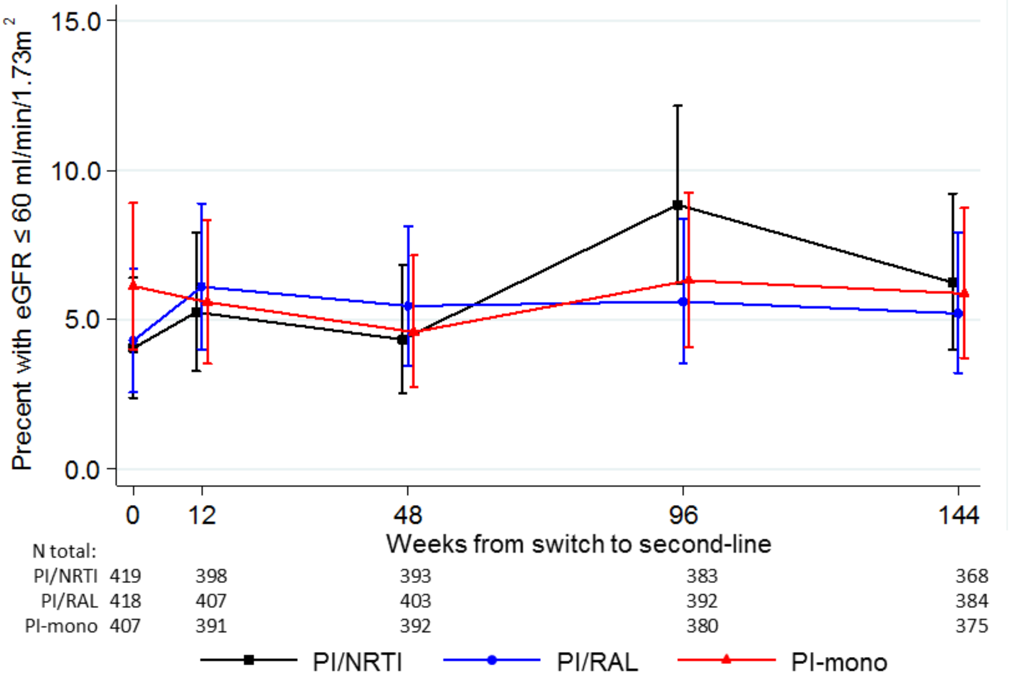

P values comparing the groups by Generalised Estimating Equations across all weeks (testing any direction of effect):
Global GEE p=0.52
PI/RAL vs PI/NRTI GEE p=0.16
PI-mono vs PI/NRTI GEE p=0.62
